# Initial antimicrobial management of sepsis: increased prehospital blood lactate levels for identifying sicker patients who may benefit from prehospital antibiotic therapy initiation

**DOI:** 10.1186/s13054-021-03801-4

**Published:** 2021-10-30

**Authors:** Romain Jouffroy, Benoît Vivien

**Affiliations:** 1 Service de Médecine Intensive Réanimation, Hôpital Universitaire Ambroise Paré, Assistance Publique – Hôpitaux de Paris, Paris Saclay University, Boulogne Billancourt, France; 2 SAMU de Paris, Service d’Anesthésie Réanimation, Hôpital Universitaire Necker - Enfants Malades, Assistance Publique - Hôpitaux de Paris, Université de Paris, Paris, France

To the Editor:

We read with great interest the recent review published in the Journal by Niederman et al. [1] emphasizing that immediate, empiric, broad-spectrum antibiotic therapy to severe sepsis and/or shock patients reduces mortality, despite the fact that it can lead to antimicrobial overuse and resistance; and should be accompanied by an early de-escalation and antimicrobial stewardship.

Early sepsis recognition and appropriate treatment initiation, mainly antibiotic therapy and hemodynamic optimization, are cornerstones for management of septic patients, especially the sicker ones, to improve prognosis [2, 3]. As underlined by Niederman et al. [1], sepsis occurs commonly outside the hospital environment in 70% of cases [4], requiring either ambulatory treatment or hospital admission for the most severe form. Nevertheless, for the latter, antibiotic therapy and hemodynamic optimization will only be initiated in the emergency department. With the exception of the most severe cases for which the diagnosis is obvious, e.g., purpura fulminans or patients with low blood pressure and skin mottling in a sepsis context, sepsis diagnose, and severity assessment remain a daily challenge in the prehospital field. For those patients, we suggest using the point of care (POC) lactate measurement. On the one hand, blood lactate level is a criterion for septic shock diagnosis and is associated with poorer outcome among septic shock patients [3]. On the other hand, POC lactate measurement devices have been validated in the prehospital environment [5]. Therefore, we suggest that a prehospital sepsis severity assessment strategy based on blood lactate level POC assessment may be helpful in identifying the sicker septic patients, i.e., those for whom, in order to reduce sepsis related mortality, prehospital antibiotic therapy instauration cannot wait until hospital admission and should be initiated from the point of prehospital care.

## Data Availability

Not applicable.
